# Data resource profile: PharmaNet, the database for prescription drug dispensing in British Columbia, Canada

**DOI:** 10.23889/ijpds.v8i6.3375

**Published:** 2026-06-18

**Authors:** Andrew Li, Yinshan Zhao, Scott D. Emerson, Kimberlyn McGrail, Sandra Peterson, Helen Tremlett

**Affiliations:** 1 Department of Anesthesiology, Pharmacology, and Therapeutics, University of British Columbia, Vancouver, BC, Canada; 2 Population Data BC, University of British Columbia, Vancouver, BC, Canada; 3 British Columbia Centre for Excellence in HIV/AIDS, Vancouver, BC, Canada; 4 School of Medicine, Simon Fraser University, Burnaby, BC, Canada; 5 Centre for Health Services and Policy Research, University of British Columbia, Vancouver, BC, Canada; 6 Faculty of Medicine (Neurology), University of British Columbia and the Djavad Mowafaghian Centre for Brain Health, Vancouver, BC, Canada

**Keywords:** administrative health data, real world data, Canada, epidemiology

## Abstract

**Introduction:**

PharmaNet is a drug information system established in late 1995 for administrative purposes and to improve patient safety in the Canadian province of British Columbia (BC). PharmaNet contains almost 30 years of data on all prescription drugs and some medical supplies dispensed at outpatient pharmacies in BC.

**Methods:**

Developed and maintained by the BC Ministry of Health, PharmaNet supports drug dispensing and helps monitor medication use. It also facilitates claims related to PharmaCare, a public programme assisting BC residents in paying for prescription medications, medical supplies, and devices. Outpatient pharmacists are required to submit information to PharmaNet for every prescription dispensed regardless of the payer.

**Results:**

Data are considered to be complete and valid from January 1st, 1996. As of December 2025, PharmaNet contained over 1.8 billion records and more than 75 million new records are added annually. Each record represents a dispensation and includes over 80 variables capturing patient demographics (e.g., age and sex), drug dispensing (e.g., drug identification number, fill date, and days supply), payment information (e.g., professional fee and dispensing fee), and information pertaining to the prescriber and pharmacy that issued the prescription. Each person is identified by their Personal Health Number, a unique lifelong identifier assigned to each BC resident for accessing healthcare. This also permits linkage with other health datasets, including physician services and hospital records. PharmaNet data can be requested for research purposes through a standard data access process and, once approved, deidentified data are released to a secure remote research environment.

**Conclusions:**

PharmaNet is a powerful resource for secondary health research. In addition to supporting studies of drug utilisation, it can when linked to other BC administrative databases enable a broad range of health research, including policy evaluation and long-term real-world drug effectiveness and safety. The data have also been used in multi-jurisdictional research through distributed analyses.

## Key features

- PharmaNet is an online, real-time prescription drug information system capturing all outpatient and community pharmacy medication dispensations in British Columbia (BC) since 1996, regardless of payer.
- PharmaNet was developed by the BC Ministry of Health to improve patient safety and for administrative purposes. However, it can be repurposed for secondary usage to support research endeavours.
- PharmaNet covers a large, diverse population of over 5.7 million residents (as of 2025) in Canada’s westernmost province, with records for all dispensations irrespective of residency or insurance status.
- Each record includes detailed information on the dispensed product or drug, patient details, prescriber and pharmacy characteristics, and financial information.
- Records are indexed using a unique, lifelong Personal Health Number, enabling linkage with other BC datasets, including administrative data.
- Deidentified PharmaNet data are available for research through a formal data access process, with secure access provided via Population Data BC or Health Data Platform BC.

## Introduction

With a population of 5.7 million, the westernmost Canadian province of British Columbia (BC) was an early pioneer in the development and linkage of routinely collected administrative data for health research through a collaboration that began more than three decades ago between the BC Ministry of Health (MoH) and the Centre for Health Services and Policy Research at the University of British Columbia. Examples of information captured by healthcare administrative databases in BC include dispensed prescription drugs, inpatient hospital day procedures in hospital, and emergency department encounters, births and deaths, cancer, and healthcare practitioner services. Other ministries and organisations outside the health sector also collect and maintain administrative data that have a health component associated with them or contain information relevant for public health. Examples include educational information from the Ministry of Education, work and income information from the Ministry of Labour, and injury related claims from WorkSafe BC.

One of the most important healthcare databases in BC is PharmaNet, developed and maintained by the MoH in late 1995 (the data were governed by the College of Pharmacists until 2005) [1]. This database includes information pertaining to prescription drugs dispensed in outpatient and community pharmacies in the province and its main purpose was to help improve patient care and safety. It helps prevent accidental duplication of prescriptions and prescription fraud, protects residents from drug interactions and dosage errors, and offers health professionals access to patients’ comprehensive medical information [1].

Beyond administrative purposes, these routinely collected data can be repurposed for secondary research use as permitted under the BC Freedom of Information and Protection of Privacy Act or the BC Statistics Act, which allow the disclosure of deidentified personal information if certain strictly enforced conditions are met, ensuring research use upholds strong privacy and ethical standards. More specifically for PharmaNet data, the e-Health Act ensures that patient privacy is protected when those data are used for research purposes. Despite its central role in pharmacoepidemiology and health services research in BC, PharmaNet’s complexity, technical nature, and lack of consolidated resources create a steep learning curve. No comprehensive resource of PharmaNet exists, representing a critical gap for researchers in pharmacoepidemiology and health services. As such, this data resource profile has three main objectives: (1) to describe the structure and content of PharmaNet; (2) to outline data quality considerations and access processes; and (3) to discuss strengths, limitations, and applications in population-based research.

## Prescription Drugs in the BC Healthcare System

Canada’s universal health insurance system – “Medicare” as it is affectionately known – provides public coverage to all Canadian residents for medically necessary physician and hospital services, including diagnostic tests regardless of their age, income, or province/territory of residence, as defined under the Canada Health Act (CHA) [2]. However, the benefits of Canada’s universal public health insurance plan as defined under the CHA do not currently extend to prescription medications. There is no national standard for public drug programmes, but each province and territory offers some form of public subsidy for prescription drugs, at their discretion.

In BC, the public programme, PharmaCare, helps BC residents pay for prescription medications, medical supplies and devices, and pharmacy services through various needs-based or catastrophic plans (see Table 1: all available plans as of 2026). These plans are universally available to all who are eligible for BC’s Medical Services Plan – the provincial health insurance programme [3] and residents can be covered by several plans simultaneously. The Fair PharmaCare Plan is the most common and its coverage is determined at the household level according to income-based deductibles and family maximums. Deductibles in BC are broadly comparable to those in other Canadian provinces, typically ranging between 0%–5% of household income for those with annual incomes below $40,000 [3, 4].

**Table 1:** Summary of the British Columbia PharmaCare Plans that Help Residents Pay for Prescription Medications, Medical Devices and Supplies, and Pharmacy Services (as of April 2026)

**Name of plan**	**Effective year**	**Description**
Long-term Care (Plan B)	1974	100% coverage for eligible prescription drugs for permanent residents of licensed long-term care facilities. Coverage is automatic upon becoming a permanent resident of a Plan B long-term care facility.
Income Assistance (Plan C)	1974	100% coverage of eligible prescription costs for BC residents who are receiving benefits and income assistance though the Ministry of Social Development and Poverty Reduction or in the care of or in agreement with the Ministry of Children and Family Services.
Children in the At Home Program (Plan F)	1989	100% coverage of eligible prescriptions for children and teens with a sever disability or complex health care needs through the At Home Program of the Ministry of Children and Family Development (MCFD). Registration is completed by MCFD.
Cystic Fibrosis (Plan D)	1995	100% or partial coverage of eligible digestive enzymes and other products on the cystic fibrosis formulary for residents registered with a provincial cystic fibrosis clinic. Registration is completed by cystic fibrosis clinic.
Psychiatric Medications (Plan G)	1997	100% coverage for certain psychiatric medications for BC residents of any age with clinical and financial need (e.g., annual income < $42,000). Coverage is up to 1 year and needs to be renewed.
BC Centre for Excellence in HIV/AIDS (Plan X)	2001	100% coverage for antiretroviral drugs for HIV-positive individuals through the BC Centre for Excellence in HIV/AIDS (Canada’s largest HIV/AIDS research and treatment facility) Drug Treatment Program.
Fair PharmaCare Plan (Plan I)	2003	Income-based plan covering many prescription drugs and dispensing fees, and some medical devices and supplies. Self registration is required. Replaced the senior plan (Plan A) and the universal plan (Plan E).
Palliative Care (Plan P)	2005	100% coverage of eligible costs for medications used in palliative care. Registration is completed by physicians or nurse practitioners.
Smoking Cessation (Plan S)	2011	100% coverage of nicotine gum, lozenges or patches, and some or all the cost of certain smoking cessation prescription drugs.
First Nations Health Benefits (Plan W)	2017	Provides 100% coverage of many prescription drugs, dispensing fees, some medical devices and supplies, and some over-the-counter drugs. Funded by First Nations Health Authority.
Assurance Plan (Plan Z)	2019	100% coverage of contraceptives and mifepristone/misoprostol, opioid use disorder treatment, and Medical Assistance in Dying medications.
Medication Management (Plan M)	2024	Covers individuals for eligible medication management services provided by pharmacies, such as clinical services, medication reviews, and publicly funded vaccinations.

Source: BC PharmaCare plans (https://www2.gov.bc.ca/gov/content/health/health-drug-coverage/pharmacare-for-bc-residents/who-we-cover).

**Figure 1:**
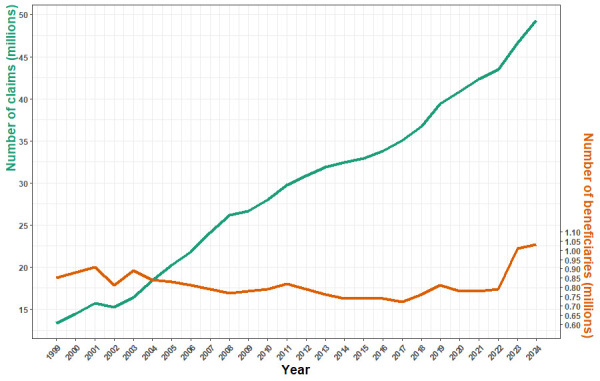
Number of PharmaCare Claims and Beneficiaries per Year (1999 to 2024)

Source: BC Ministry of Health PharmaCare Trends report (https://www2.gov.bc.ca/gov/content/health/practitioner-professional-resources/pharmacare/pharmacare-publications).

PharmaNet is BC’s drug information, claims adjudication and payment system that accompanies the BC PharmaCare programme. Every prescription dispensation performed at an outpatient or community pharmacy in the province goes through PharmaNet, creating a single database containing the prescription medication history for all BC residents regardless of prescriber, pharmacy, or payer. Every record within the database corresponds to a unique dispensation. As of December 2025, PharmaNet contained over 1.8 billion rows and more than 75 million new rows are added every year [1, 5]. Figure 1 shows the number of annual PharmaCare claims and beneficiaries from 1999 to 2023.

## Methods

**Figure 2:**
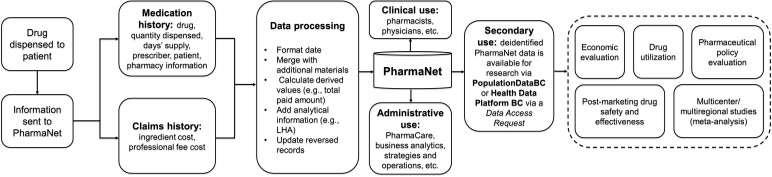
Conceptual Framework Illustrating the Data Flow from Community Pharmacies into PharmaNet and its Subsequent Administrative and Secondary Research Uses

Data in PharmaNet are considered to be complete and valid from January 1^st^, 1996. It captures almost every dispensation occurring in BC outpatient or community pharmacies including those for non-BC residents, privately paid prescriptions and non-PharmaCare payments. Pharmacists are required to submit prescription information to PharmaNet electronically in real-time at the point of dispensation (Figure 2).

However, prescriptions dispensed for less than 5% of the BC population are not captured due to federal government plan exclusions, including First Nations residents covered by the Health Canada’s Non-Insured Health Benefits program prior to 2013, federal prisoners, military personnel, and members of the Royal Canadian Mounted Police prior to early 2013. In addition, there are four instances when dispensed drugs are not captured in the database: (1) medications used in hospitals (e.g., during inpatient care or emergency department use) or at designated mental health centres; (2) sample medication from a physician’s office; (3) medications dispensed through BC Cancer Agency (e.g., oncology medications such as chemotherapy drugs are not included, but drugs to help mitigate adverse effects of cancer treatment are included), BC Transplant Society, BC Renal Agency, and BC Centre for Excellence in HIV/AIDS before 2020 (antiretroviral drugs for the treatment of HIV); and (4) special programmes administered by BC’s Provincial Health Services Authority (e.g., a Retinal Disease Program). Data for these specialised populations and settings are available but requires additional permission from their respective programmes (e.g., BC Cancer Agency for oncology research).

PharmaNet is a relational database maintained by the BC MoH, consisting of seven tables that together capture the complete dispensing and reimbursement record for each transaction. The tables are in long format, whereby each dispensation is recorded as a separate row, therefore an individual will have multiple rows of data. The core table contains merged records between PharmaCare Claims and Dispensing tables, forming the foundation for most research queries. Supporting tables provide additional context: health product information identifying authorised drugs; benefit plan and account accumulator tables that capture how claims were adjudicated and reimbursed under PharmaCare; a payment status table classifying each transaction as paid, unpaid, or reversed; a professional services table; and a health product extract for substance use disorder medications. Records are linked across tables via common identifiers, enabling the construction of longitudinal medication histories at the individual patient level.

## Results

### Contents

Since late 1995, dispensations at BC community pharmacies along with information associated with these transactions are transmitted to PharmaNet. Each patient is identified by their Personal Health Number (PHN), a unique lifelong identifier assigned to each BC resident enrolled in the Medical Services Plan that follows the patient across health service sectors. As of 2025, each dispensation record corresponds to over 80 variables routinely available for research use, and 4 variables subjected to special approval. Table 2 provides an overview of the main variables. Complete documentation is searchable online: (https://www.popdata.bc.ca/sites/default/files/documents/ data/Core%20and%20Non-Core/PNET_All_Available_ Variables_Jan_23_2024.pdf).

**Table 2:** Key Variables Commonly Used for Research in PharmaNet

**Description**	**Explanation**
**Patient details**	
Personal Health Identifier	Deidentified Personal Health Identifier, a unique lifelong identifier of BC residents.
Age	Age is calculated using the Event/Service date and the birthdate in the reference data
Birthdate	Date individual was born, only month and year available
Sex	Sex of the individual
Local Health Area	Client residential Local Health Area (a lower level of region created by the BC MoH for administrative and planning purposes)
Postal Code	Residential 6-digit postal code available upon special approval
**Dispensing details**	
Date	Date when treatment, product, or service was provided to the client
Product label	The Drug Identification Number/Product identification Number* assigned by Health Canada, used to uniquely identify a particular drug by chemical, dosage, form, and manufacturer
Days’ supply	Prorated amount of the prescription based on the allowed days supply
**Financial details**	
PharmaCare cost	Total amount paid by PharmaCare to the pharmacy for the drug cost and pharmacy fee
Total cost	Total billed amount (calculated as billed ingredient cost + billed professional fee + billed special service fee)
Professional fee	Amount of the professional fee paid to the facility (usually a pharmacy) that submitted the claim
**Other details**	
Drug plan	PharmaCare plan (some plans have been used as proxies for sociodemographic characteristics; see Strengths section)
Practitioner type	Practitioner type (e.g., physician, dentist, nurse practitioner, podiatrist, midwife, veterinarian, pharmacist)
Pharmacy	Deidentified Pharmacy ID, assigned by the Canadian Pharmaceutical Association

*Product Identification Numbers are assigned by PharmaCare when a Drug Identification Number has not been assigned or a separate identifier is needed.

Researchers can request access to important variables including patient demographics (e.g., age, sex, and a unique study ID [i.e., deidentified PHN, enabling connection of records for the same person within the PharmaNet dataset and across other linkable datasets]); date of dispensation; information related to the prescription dispensed such as drug name, dispensed quantity, days supply, Drug Identification Number (DIN); payment information such as drug cost, cost portions covered by PharmaCare and other payers, and pharmacist professional fee; information on the prescriber and pharmacy; and professional services provided. In PharmaNet, the dispensed drug is identified by a unique DIN, a computer-generated eight-digit number assigned by Health Canada to a drug product prior to being marketed in Canada [6] and searchable at [https://health-products.canada.ca/dpd-bdpp/]; a Product Identification Number (PIN) assigned by PharmaCare when a DIN has not been assigned; or a separate identifier is needed for PharmaCare purposes (e.g., when pain medication is used for opioid use disorder, the PIN is used, when used for pain management, DIN is used [7]) [8]. The DINs are mapped to the Anatomical Therapeutic Chemical (ATC) code, a hierarchical classification system designed by the World Health Organization and searchable at [https://atcddd.fhi.no/atc_ddd_index/] [9].

### Data quality

PharmaNet is maintained by the MoH and the tables are updated weekly. However, due to reversals, data are not considered stable for at least three months after the event [10]. Therefore, a three month latency period is recommended when defining study periods to ensure data stability and accuracy [10]. Data between 1985 to 1995 are also available, but these earlier data only capture dispensations paid for through PharmaCare plans captured in effect at that time.

There are some data quality issues that researchers should note. While PharmaNet was designed to be comprehensive, the accuracy of each individual record still depends on data entry at the pharmacy level. As such, occasional discrepancies may occur if a prescription is omitted or if an entry is made for a prescription that was not actually dispensed [10]. These types of errors are expected but infrequent as data quality is supported through audits and system monitoring [11]. While pharmacy level entry errors are not addressable at the researcher level, they are expected to be infrequent given the audits and system monitoring in place. Another notable limitation in data quality is the lack of system safeguards to ensure the consistency between dispensed quantity and dispensed days’ supply recorded on a prescription [10]. For example, in practice, a prescription can be entered as a 30-day supply, but the corresponding tablet count may not align with the expected quantity for that duration and dosing frequency. Such discrepancies are not automatically flagged, and since days’ supply is calculated by the pharmacist, it is more susceptible to error. Researchers are therefore advised to use quantity dispensed.

## Discussion

### Data Resource Use

PharmaNet has been used extensively to answer research questions [12] either as a stand-alone resource for basic analyses of drug utilisation and spending, or linked with other administrative databases to support population-based studies. Linkage is possible due to an individual’s unique lifelong PHN assigned to all BC residents. For secondary use purposes, this number is deidentified but can still be used for data linkage. Policy makers have taken advantage of PharmaNet data linked with other health databases to evaluate the effectiveness, safety and cost of health policies implemented within the province. PharmaNet is also a powerful source for assessing real-world drug effectiveness and safety. In particular, we highlight three areas: firstly, because PharmaNet has almost 30 years of data, it is possible to study long-term medication use and adverse drug events with prolonged latency periods. Secondly, its population-wide coverage captures virtually all outpatient medication dispensations in BC, irrespective of payer, allowing for subpopulation analyses and a broader view of medication use in the province. Finally, through collaboration across Canada and internationally, PharmaNet data has been used in large observational studies, such as those conducted by the Canadian Network for Observational Drug Effect Studies [13]. Table 3 presents a selection of recently published studies utilising PharmaNet data, to illustrate how different research questions shape study design and analytical approach.

**Table 3:** Examples of published studies using PharmaNet data

**Author (year)**	**Cohort definition**	**Statistical analysis**	**Main findings**
**Pharmaceutical policy**			
Fisher (2022) [16]	Incident users of infliximab with inflammatory conditions; stratified into four cohorts (policy + 3 historical); 365-day follow-up.	Cumulative incidence of infliximab refills, switching to biologics, use of additional health services via log-likelihood ratio test.	Mandatory nonmedical switching policy from originator to its biosimilar resulted in decrease in refills and did not increase health services utilisation.
Carney (2021) [17]	Incident users of smoking cessation pharmacotherapies (varenicline, bupropion, NRT), 1-year follow-up.	Propensity score-adjusted log-binomial regression comparing cardiovascular and neuropsychiatric hospitalisations and mortality across therapies.	With the Smoking Cessation Program, varenicline was associated with fewer adverse events compared to NRT and bupropion was comparable to NRT.
Schummers (2025) [18]	Female residents aged 15-49; pre/post universal contraception coverage policy (April 2021-June 2024).	Controlled time series with segmented regression; synthetic control derived from nine Canadian provinces.	Universal no-cost contraception coverage policy increased prescription contraception use, driven largely by increased LARC uptake.
Sharma (2025) [19]	Low-income households (<$13,750) pre/post copayment elimination policy (2017–2021); high-income households (>$45,000) as control).	Controlled interrupted time series comparing prescription expenditures and dispensing between intervention and control cohorts.	Elimination of copayments for low-income households led to significant increase in prescription drug expenditures and dispensing, improving medication access for low-income households.
**Drug utilisation (e.g., trends, adherence, and polypharmacy)**			
Nitschke (2025) [20]	Pregnant people with deliveries (2000–2021) with dispensed ADHD stimulants; followed from 1 year preconception to 1 year postpartum.	Prevalence trends by medication type and age group; patterns of use and discontinuation assessed longitudinally.	11-fold increase in prenatal ADHD stimulant use over the study period, with 77% discontinuing before or during pregnancy.
Salmasi (2024) [21]	Incident OAC users with AF; followed for median 6.7 years from OAC initiation.	PDC calculated per 90-window as time-dependent covariate; generalised mixed effect linear regression identifying factors associated with adherence.	Over half of AF patients were non-adherent to OAC therapy. Adherence to VKA declined over time while COAC adherence increased slightly.
Chertcoff (2024) [22]	Residents with MS in 2017; polypharmacy defined as ≥5 concurrent medications for >30 consecutive days.	Polypharmacy prevalence was estimated cross-sectionally; logistic regression identifying patient factors associated with polypharmacy.	>1 in 4 MS patients met criteria for polypharmacy, with higher odds among women, older individuals, and those with more comorbidities, but lower SES.
**Health Economics**			
Khakban (2023) [23]	Residents with MS (2001–2020) matched 1:5 to controls without MS on sex, age, and cohort entry date.	GLMs estimating excess direct medical costs (inpatient, outpatient, medications) between MS patients and matched controls.	Excess cost of MS was $6,881 per patient-year, with medications (particularly DMTs) accounting for 65% of excess cost.
Itiola (2024) [24]	Adults ≥18 with diabetes and ≥ PharmaCare-eligible blood glucose test strip claim (2013–2019).	Interrupted time series estimating cost savings and policy adherence.	Quantity prescriptions on blood glucose test strip coverage led to $44.6 million in savings without increasing health service utilisation.
**Long-term exposure and adverse events**			
Canney (2025) [25]	Patients with glomerular disease (IgA nephropathy, focal segmental glomerulosclerosis, membranous nephropathy, and minimal change disease).	Cox regression models estimating association between cumulative immunosuppressive medications exposure and composite cardiovascular outcomes.	Cumulative calcineurin inhibitor and cyclophosphamide exposure associated with significantly higher cardiovascular event risk; corticosteroids were not.
Graf (2024) [26]	Residents with MS (1996–2017); DMD-exposed vs. unexposed periods compared within individuals.	Negative binomial and proportional means regression estimating association between DMD exposure and infection-related hospitalisations, physician claims, and prescription fills.	DMD exposure associated with lower infection-related hospitalisations and physician visits but higher prescription fills.
**Multicenter/multiregional studies**			
Filion (2020) [27]	New users of SGLT2 inhibitors matched to DPP-4 inhibitor users on time-conditional propensity scores across 7 Canadian provinces and UK (2013-2018).	Cox proportional hazards models estimating site-specific hazard ratios; pooled via random effects meta-analysis across jurisdictions.	SGLT2 inhibitors associated with significantly lower risk of MACE, heart failure, and all-cause mortality compared to DPP-4 inhibitors.
Fisher (2022) [28]	Pregnant women with pregnancies ending in live birth, stillbirth, or abortion across 5 Canadian provinces, UK, and US (2002–2014).	Population-based cohort comparing prevalence and trends of antiemetic use across jurisdictions using pharmacy claims and prescription data.	Antiemetic use during pregnancy increased across all jurisdictions; Canada had highest prevalence, with doxylamine/pyridoxine predominating vs. ondansetron in the US.
Ng (2024) [29]	People with MS across 4 Canadian provinces (1996-2017); followed from first MS/demyelinating disease event until emigration, death, end of follow-up.	Recurrent events proportional means models for infection-related outcomes; stratified multivariate Cox proportional hazards models for 15 categories of incident adverse events by DMD type.	Most DMDs associated with lower infection-related hospitalisations; second-generation DMDs, particularly alemtuzumab, associated with substantially elevated risks of thyroid disorders, hypertension, and cardiovascular disease.

Abbreviations: NRT, Nicotine Replacement Therapy; LARC, Long-Acting Reversible Contraception; BZD, benzodiazepines; ADHD, Attention-deficit/hyperactivity disorder; OAC, oral anticoagulant; PDC, proportion of days covered; AF, atrial fibrillation; GLM, generalised linear model; MS, multiple sclerosis; DMT, Disease-Modifying Therapies; DMD, disease-modifying drugs; SGLT2, sodium glucose cotransporter 2; MACE, Major Adverse Cardiovascular Events; DPP-4, dipeptidyl peptidase-4.

Additionally, we provide two applied examples illustrating how researchers have utilised PharmaNet data. Firstly, to investigate changes in sedative use in BC, a drug utilisation study was conducted [14]. The cohort was mainly derived from PharmaNet. BC residents aged ≥13 with ≥1 dispensation of a Z-drugs or benzodiazepines, were identified via DIN or ATC code. PharmaNet, a dispensation database, lacks diagnostic information. Therefore, linkage to other administrative databases was required to apply clinical exclusions (e.g., persons with schizophrenia or bipolar disorder). Drug utilisation was quantified through monthly prevalence (number of individuals with an active dispensation in each month) and 6-month incidence rates (new users with no prior dispensation in the preceding 5 years). This example demonstrates PharmaNet’s capabilities to provide population-level drug utilisation insights. Secondly, to investigate whether use of diabetic pharmacotherapy increases the risk of endometrial cancer, a case-control study was carried out [15]. Cases were identified from the BC Cancer Registry and matched to population controls. Data on the use of diabetic pharmacotherapy were obtained from PharmaNet. The cancer registry dataset was linked to MSP registration using a probabilistic linkage based on Personal Health Number, last name, and date of birth. Risks with use of individual drugs and combination treatment were estimated using conditional logistic regression, finding that metformin was not associated with a reduced risk of endometrial cancer compared to other medications.

### Strengths

The design and implementation of PharmaNet was remarkably forward thinking, resulting in one of the oldest and most comprehensive prescription drug recording systems in Canada providing almost 30 years of provincial coverage. In Canada, only Saskatchewan (since 1990) and Manitoba (since 1995) have comparable single, centralised systems capturing virtually all dispensation data, though both serve considerably smaller populations [30, 31]. Alberta’s Pharmaceutical Information Network (established 2008) captures all prescription drugs but does not record financial information [32]. Researchers from Ontario and Québec have access to data from the Ontario Drug Benefit (ODB) programme and the Régie de l’assurance maladie du Québec (RAMQ) plan, respectively. Both are less comprehensive as they cover only specific population subgroups such as older adults, children and teenagers, and social assistance recipients [33, 34]. Data from the RAMQ has been shown to over-represent individuals of lower socioeconomic status, and therefore the conclusions and inferences drawn are considered to be less generalizable [35]. Finally, the Atlantic provinces maintain separate databases with varying coverage [36–39]. PharmaNet is compared to other provincial dispensation databases in Table 4.

**Table 4:** Comparison of the Different dispensation Registries in Canadian Provinces

**Province**	**Data holder**	**Drug information System**	**starting year**	Population coverage (million)*	**Drug coverage**	**Exclusions**
British Columbia (BC)	BC Ministry of Health	PharmaNet	1996	5.72	All prescription drugs	Inpatient hospital or emergency department administered drugs Samples from doctors’ office Medications dispensed through: - BC Cancer Agency - BC Transplant Society - BC Renal Agency - BC Centre for Excellence in HIV/AIDS
Alberta (AB)	AB Health	Pharmaceutical Information Network	2008	4.96	All prescription drugs	Drugs administered in hospitals and health care facilities
Saskatchewan (SK)	SK Health	Prescription Drug Plan	1990	1.25	All prescription drugs	Hospital inpatient medication Saskatchewan Cancer Agency
Manitoba (MB)	MB Health	Drug Program Information Network	1995	1.50	All prescription drugs	Hospital pharmacies Nursing stations Ward stock CancerCare Manitoba
Ontario (ON)	ON Ministry of Health	Ontario Drug Benefit (ODB) programme	1996	4.0^†^	Reimbursed prescription drugs via the ODB program	Drugs administered in hospitals and health care facilities
Québec (QC)	Régie de l’assurance maladie du Québec (RAMQ)	Régie de l’assurance maladie du Québec	1997	3.9^†^	Reimbursed prescription drugs via the RAMQ plan	Drugs administered in hospitals and health care facilities
New Brunswick (NB)	NB Department of Health	NB Drug Information System	2008	0.85	All prescription drugs	Drugs administered in hospitals and health care facilities
Newfoundland and Labrador (NL)	NL Health Services	NL Pharmacy Network	2009	0.54	All prescription drugs	Hospital outpatient medication not covered by NLPN (NLPN contains information from three hospital outpatient dispensaries) Hospital inpatient medications
Nova Scotia (NS)	NS Department of Health and Wellness	NS Drug Information System	2016	1.07	All prescriptions drugs	Hospital inpatient & outpatient medication Prescriptions for long-term care facilities not covered by a pharmacy Prescriptions dispensed at Canadian Forces pharmacies or correctional centres not serviced by a community pharmacy
Prince Edward Island (PEI)	Health PEI	Drug Information System	2008	0.18	All prescriptions drugs	Drugs administered in hospitals and health care facilities

^*^Population coverage is based on population estimates provided by Statistics Canada as of the first quarter of 2025.

^†^Population coverage for Ontario and Québec reflects the estimated number of residents covered under the ODB programme and RAMQ plan respectively, rather than total provincial population estimates, as these programmes cover specific population subgroups only.

Internationally, PharmaNet coverage is comparable to prescription registries in countries such as Finland, Denmark and Sweden, which were established in 1994, 1995 and 2001 respectively [40–42]. Similarly to these Nordic countries, loss to follow-up is only limited to residents that move out of the jurisdiction [40–42]. This is in contrast to data from pharmacy systems of health maintenance organisations in the United States which, although spanning decades, have substantial annual turnover in enrollment [43]. In comparison to prescribing databases (i.e. containing written prescriptions), such as the United Kingdom Clinical Practice Research Datalink and the Health Improvement Network [44, 45], PharmaNet, based on dispensed medication, provides a better proxy of consumed drugs, but cannot say anything about prescriptions that were never filled.

PharmaNet has several unique strengths. Firstly, PharmaNet has demonstrated an impact on improving appropriate medication usage. For example, its implementation was associated with a dramatic reduction in inappropriately filled prescriptions for opioids and benzodiazepines [46]. In addition, the inclusion of PharmaCare plan information within PharmaNet allows researchers to infer certain characteristics based on the plan under which the prescriptions were paid. For example, Plan C (Income Assistance) has been used as a proxy for low socioeconomic status [47–49], while Plan B (Long-term Care) can be used to identify individuals residing in residential care settings [50]. Other plan types, such as Plan S (Smoking Cessation), may offer opportunities to identify smoking-related information that is otherwise unavailable in other data sources.

### Limitations

A limitation of PharmaNet is that gaps in prescription medication history are possible because drugs dispensed in inpatient hospital settings, emergency departments, and out-of-province are not captured. Consequently, in pharmacoepidemiologic studies, this period is considered as “immeasurable” because the subject cannot be recognised as being exposed to a certain drug. If this gap exists during follow-up for a cohort study or prior to the index date for a case-control study, the analysis may be susceptible to immeasurable time bias whereby the benefit of a drug is distorted [51]. Immeasurable time bias can be minimised with appropriate methodological approaches and careful study design [52, 53].

Over-the-counter medications, such as aspirin (acetylsalicylic acid), and samples provided by a physician are not recorded in PharmaNet. Additionally, records in PharmaNet represent drugs that have been dispensed but there is no ability to track actual consumption, which results in potential misclassification for drug exposure. However, medication adherence studies have found prescription data to be a relatively valid proxy for actual medication use [54]. PharmaNet dispensation data in particular were found to accurately reflect medication adherence in heart failure patients [55]. Meanwhile, another study found that the accuracy of prescription medications was generally high, but varied by medication type. For example, insulin and warfarin suffered relatively higher rates of dose and frequency errors [56]. Well-designed future studies to comprehensively evaluate the accuracy and validity of PharmaNet data would help support its use for clinical and research purposes.

Another limitation is that a single dispensation of an expensive drug may appear as multiple records in PharmaNet. The maximum drug cost per claim is $9,999.99 CAD, so any dispensation exceeding this amount is split into separate claims with the drug cost, dispensed quantity, and days’ supply divided proportionally [57]. For example, a drug with a 30-day supply that cost $27,000 would be split into three records of $9,000 each, with ten dispensed quantity and days’ supply per record. When working with expensive drugs, analysts should count only unique dispensation records to avoid overcounting.

Finally, as with other administrative databases, PharmaNet does not have information on important factors that may be potential confounders in analyses; these include lifestyle information (e.g., smoking, exercise, and diet), environmental exposures, family medical histories, and social determinants of health information (e.g., education, occupation).

## Data Access

The primary users of PharmaNet are health professionals (pharmacists, physicians, and nurse practitioners) and MoH employees, but academic researchers can request access via, for example, a Data Access Request (DAR) with Population Data BC or Health Data Platform BC (HDPBC). Population Data BC is a multi-university resource that provides researchers in BC deidentified, longitudinal, individual-level health administrative data for BC residents to support health-related research (https://www.popdata.bc.ca/) [58]. The HDPBC is a data access programme that supports case-level and population-level research within the health sector for academics by offering streamlined access to health data (https://healthdataplatformbc.ca/) [5]. Most of the contents within PharmaNet are available for research, but some claims information requires additional approval, for example Plan W (First Nations Health Benefits), Plan Z (mifepristone and misoprostol for abortions), and Plan X (antiretroviral drugs dispensed from the BC Centre for Excellence in HIV/AIDS).

A DAR is a formal document that requests access to data from the Data Steward(s) responsible for maintaining the databases. A successful DAR will clearly outline research objectives, methodology, and specify in detail, the data needed for the research project, including a study population definition and a complete list of data files, and years of data required. Each DAR is assessed on its own merits against a standardised criteria by the responsible Data Steward(s). The studies also need to obtain ethics approval and have undergone peer review. Population Data BC facilitates the DAR submission but is not involved in any adjudication processes. A successful DAR takes time, planning, and attention to detail. Access to the data will be denied if the DAR application is inadequate or incomplete.

Upon approval, data can be accessed in a Secure Research Environment (SRE) through Population Data BC or in a Trusted Analysis Environment (TAE-BC) through HDPBC, both of which come with a large suite of analytical software. The SRE and TAE-BC can only be accessed within Canada and no record-level data or results constituting small cells are permitted to be transferred outside the SRE or the TAE-BC. Complete documentation for data access can be found online: (https://www.popdata.bc.ca/data_access).

## Conclusion

PharmaNet is a significant resource for research that relates to prescription drugs because of its long history, comprehensiveness, and inclusion of dispensation information regardless of payer. The database is nonetheless complex and require careful attention from researchers to ensure appropriate use and high-quality research findings.

## Data Availability

PharmaNet administrative health data cannot be publicly shared because of BC MoH policies regarding data privacy and security. The data contains potentially identifying and sensitive patient information. All data presented in this manuscript are summary level data that can be publicly accessed. For researchers, requests for the data can be made as described in the data access subsection.
